# Assessment of dizziness among older patients at a family practice clinic: a chart audit study

**DOI:** 10.1186/1471-2296-6-2

**Published:** 2005-01-06

**Authors:** Eugene CK Kwong, Nicholas JG Pimlott

**Affiliations:** 1 Department of Family and Community Medicine, University of Toronto, Toronto, Ontario, Canada; 2 Department of Family and Community Medicine, Women's College Campus, Sunnybrook and Women's College Health Sciences Center, 60 Grosvenor Street, Toronto, Ontario, M5S 1B6, Canada

## Abstract

**Background:**

Dizziness is a common complaint among the elderly with a prevalence of over 30% in people over the age of 65. Although it is a common problem the assessment and management of dizziness in the elderly is challenging for family physicians. There is little published research which assesses the quality of dizziness assessment and management by family physicians.

**Methods:**

We conducted a retrospective, chart audit study of patients with dizziness attending the Sunnybrook Family Practice Center of Sunnybrook and Women's College Health Sciences Center (SWCHSC) in Toronto. We audited a random sample of 50 charts of patients from 310 eligible charts. Quality indicators across all dizziness subtypes were assessed. These quality indicators included: onset and course of symptoms; symptoms in patients' own words; number of medications used; postural blood pressure changes; symptoms of depression or anxiety; falls; syncope; diagnosis; outcome; specialty referrals. Quality indicators specific to each dizziness subtype were also audited.

**Results:**

310 charts satisfied inclusion criteria with 20 charts excluded and 50 charts were randomly generated. Documentation of key quality indicators in the management of dizziness was sub-optimal. Charts documenting patients' dizziness symptoms in their own words were more likely to have a clinical diagnosis compared to charts without (P = 0.002).

**Conclusions:**

Documentation of selected key quality indicators could be improved, especially that of patients' symptoms in their own words.

## Background

Dizziness is a common complaint among the elderly, with a prevalence of more than 30% in people over age 65 [1] and it accounts for 2% of consultations in the primary care setting [2]. Drachman and Hart [3] described four subtypes of dizziness: vertigo, lightheadedness, dysequilibrium, and others. Several recent community-based studies of dizziness shows that, among the 4 dizziness subtypes, the proportion of vertigo was more uniform, ranging from 28 to 32% [1,4-6]. Reported frequencies of specific diagnoses for dizziness varies widely however, depending on: 1) clinical setting (primary care setting, referral center or emergency department); 2) patient age or patient populations examined; and 3) investigator bias. These methodological problems limit the generalizability of the etiological studies [3]. Kroenke et al [7] found in their systematic review that dizziness was attributed to peripheral vestibulopathy in 44%, central vestibulopathy in 11%, psychiatric causes in 16%, other conditions in 26%, and an unknown cause in 13% of cases.

Life-threatening illness is rare in patients with dizziness (with cerebrovascular disease accounting for 6%, cardiac arrythmia for 1.5% and brain tumor for <1%) [7]. However, many do have serious functional impairment, such as increased risks for falls and increased incidence of symptom-related fears, anxiety or depression [8-10]. Many patients with chronic dizziness, particularly the elderly, are under-referred for specialist consultation and thus are not receiving timely treatment [5].

When assessing dizziness, what concerns a family physician most are: 1) how to distinguish serious causes of dizziness from less urgent ones; 2) how to manage patients with chronic but yet debilitating dizziness; and 3) how to decide on the right timing and the appropriate specialty for referral. However, many family doctors describe dizziness as "confusing" and "discouraging" problem [8] and expensive investigations like electro-nystagmography and MRI are rarely helpful [4]. In fact, a diagnosis cannot be ascertained in many patients with dizziness and many patients may have more than one diagnosis [11], making management difficult. To date, there are no evidence-based guidelines in the management of dizziness among elderly patients in a primary care setting because most past studies on dizziness have been retrospective or in referral settings.

Traditionally, the approach to dizziness is "disease-oriented", in which the clinician aims, at a minimum, to exclude potentially fatal causes and possibly to diagnose a specific cause for treatment. On the other hand, some authors like Tinetti et al [12] and Kao et al [13] regard dizziness in the elderly as a "geriatric syndrome", because it represents dysfunction in more than one body system and has multiple predisposing risk factors. This function-oriented approach focuses on impairment reduction to reduce morbidity associated with dizziness, regardless of etiology. Tinetti's epidemiological population-based study [12] found that seven characteristics were associated with dizziness in the elderly: anxiety, depression, using five or more medications, impaired balance, past myocardial infarction, postural hypotension, and impaired hearing.

Despite differences in the above two approaches, both share in common certain key quality indicators as reflected in recent studies and reviews [4,7,11-15]. The purpose of this chart audit study was to assess the extent to which family physicians included these key quality indicators when assessing and managing the dizzy elderly patient.

## Methods

A retrospective chart audit was conducted at the Family Practice Center (Sunnybrook Campus) of Sunnybrook and Women's College Hospital Health Sciences Center. Inclusion criteria for the chart audit were: 1) Patients with a International Classification of Disease (ICD-9) diagnostic billing code of "780" (dizziness); 2) Patients seen between Feb 1^st ^2001 and Jan 31^st ^2003; 3) Patients 65 years of age or older when seen. Exclusion criteria were: 1) Patients who are discharged from service or died; 2) Patients whose presenting symptoms were not dizziness or any of its subtypes.

A chart audit intake form was designed [Additional File 1], which included quality indicators important in the diagnosis and management of dizziness, based on recommendations from several recent review articles and peer-reviewed studies [4,7,11-15]. A random sample from the eligible charts was then audited and the data analyzed for descriptive statistics using SPSS.

The general outcome measures/quality indicators across all dizziness subtypes include the documentation of : 1) onset and course of symptoms; 2) symptoms in the patients' own words; 3) number of medications used; 4) postural blood pressure changes; 5) symptoms of depression or anxiety; 6) falls; 7) syncope; 8) diagnosis; 9) outcome of dizziness; 10) specialty referrals.

The quality indicators specific to vertigo include the documentation of 1) episode duration; 2) relationship to head turning; 3) tinnitus and hearing loss; 4) ear examination; 5) neurological examination; 6) spontaneous nystagmus; 7) positional nystagmus (Hallpike manoevre) (16). These patients were also audited for whether audiometry was ordered and whether Epley's manoevre [17] was offered if BPV was diagnosed.

The quality indicators specific to lightheadedness include the documentation of 1) relationship to postural change; 2) cardiac symptoms; 3) syncope; 4) orthostatic blood pressure changes. These patients were also audited for whether ECG or Holter monitoring were ordered.

The quality indicators specfic to disequilibrium include the documentation of

1) falls; 2) neurological exam; 3) cerebellar signs; 4) Gait examination; 5) Romberg's sign; 6) visual acuity.

The quality indicators specifically to other non-classifiable dizziness include the documentation of symptoms of depression and anxiety.

## Results

310 charts satisfied the inclusion criteria with 20 charts excluded. A random sample of 50 charts were generated for the audit. The demographics of the sample, including age, gender living situation, are described in Table 1. Of note is that 62% of the patients are 80 years of age or older and 28% of patients are living alone.

**Table 1:** Demographics and living situation of patients (n = 50)

	Total	% Male	% Female
Age > or = 65	100% (n = 50)	42% (n = 21)	58% (n = 29)
Age < 80	38% (n = 19)	16% (n = 8)	22% (n = 11)
Age > or = 80	62% (n = 31)	26% (n = 13)	36% (n = 18)
Age range	65–91	66 to 89	65 to 91
Living Alone	28% (n = 14)	2% (n = 1)	26% (n = 13)
Living with Spouse	18% (n = 9)	12% (n = 6)	6% (n = 3)
Living with Family	8% (n = 4)	2% (n = 1)	6% (n = 3)
Living situation Not Documented	46% (n = 23)	26% (n = 13)	20% (n = 10)

The distribution of different subtypes of dizziness is described in Table 2, with more patients presenting with lightheadedness (40%) and dysequilibrium (38%) than vertigo (28%). There are more females than males among the patients with lightheadedness (30% vs. 10%) and vertigo (16% vs. 10%) whereas the ratio of females to males is roughly the same among those with dysequilibrium (20% vs. 18%). 30% (n = 15) of patients presented with more than one subtype of dizziness.

**Table 2:** Symptomatology distribution of patients

**Dizziness Subtype**	Yes			No	Not Documented
	Total	Female	Male	Total	Total

Vertigo	26% (n = 13)	16%	10%	26% (n = 13)	48% (n = 24)
Lightheadedness	40% (n = 20)	30%	10%	10% (n = 5)	50% (n = 25)
Disequilibrium	38% (n = 19)	20%	18%	6% (n = 3)	56% (n = 28)
Others	26% (n = 13)	12%	14%	6% (n = 3)	68% (n = 34)

The onset and diagnoses of dizziness are described in Table 3. 70% of patients have a precipitating factor, the commonest ones being postural change, movement and head turning. 46% of patients have no diagnosis while 10% of patients have more than one diagnoses. Among patients with an ascertained diagnosis, the most common ones are BPV (12%), labyrinthitis (10%) and TIA/Stroke (8%). Significantly, patients were more likely to be diagnosed if their symptoms were documented in their own words compared to those without such documentation (see Table 4).

**Table 3:** Onset and diagnoses of dizziness

		Percentage of Patients
**Onset**	Spontaeous	4% (n = 2)
	Precipitating Factors present	70% (n = 35)
	Postural change	38% (n = 19)
	Head turning	12% (n= 6)
	Any movement	18% (n= 9)
	Walking	10% (n = 5)
	Anxiety	2% (n = 1)
	Other factors	26% (n = 13)
	Not Documented	26% (n = 13)
		
**Diagnosis**	No	46% (n = 23)
	Yes	54% (n = 27)
	BPV	12% (n = 6)
	Labyrinthitis	10% (n = 5)
	TIA/Stroke	8% (n = 4)
	Hypertension	6% (n = 3)
	Depression/Anxiety	6% (n = 3)
	Arrhythmia	4% (n = 2)
	Alcohol	4% (n = 2)
	Dehydration	4% (n = 2)
	Others	4% (n = 2)
	More than One Diagnoses	10% (n = 5)

**Table 4:** Documentation of patients' symptoms in their own words

Documentation	With Diagnosis	Without Diagnosis
Yes (n = 22)	82%* (n = 18)	18% (n = 4)
No (n = 28)	32% (n = 9)	68% (n = 19)

*statistically significant (P = 0.002)

The documentation of general and dizziness subtype-specific quality indicators in history and physical examination are described in Table 5. It also was observed that: 1) 60% of all patients were taking at least 5 medications; 2) three vertiginous patients with associated ear symptoms were not offered audiometry; 3) none of the four vertiginous patients with an abnormal Hallpike test were documented to be treated by Epley's manoevre; 4) in the lightheadedness subgroup, ECG was ordered in only 40% and Holter monitoring in only 30% of patients.

**Table 5:** Documentation of general and dizziness subtype-specific quality indicators in history and physical examination for Patients with Vertigo, Lightheadedness, Disequilibrium and Others

		**Yes**	**No**	**Not Documented**
General (n = 50)	Postural BP changes	6%	44%	50%
	Associated Depression	6%	8%	86%
	Associated Anxiety	14%	0%	86%
	Falls	6%	30%	64%
	Syncope	2%	36%	62%
Vertigo (n = 13)	Episode Duration	38%	N/A	62%
	Relationship to Head Turning	38%	8%	54%
	Tinnitus	23%	54%	23%
	Hearing Loss	0%	0%	100%
	Ear Examination	39% (Normal:31%) (Abnormal:8%)	N/A	61%
	Neurological Exam	92%	N/A	8%
	Spontaneous Nystagmus	46% (Normal 38%) (Abnormal 8%)	N/A	54%
	Hallpike Manoevre	39% (Normal 8%) (Abnormal 31%)	N/A	61%
				
Lightheadedness (n = 20)	Relationship to Postural change	55%	15%	30%
	Chest Pain	5%	60%	35%
	Palpitation	5%	55%	40%
	Syncope	5%	50%	45%
	Orthostatic drop in BP	10%	55%	35%
	Orthostatic rise in pulse	0%	15%	85%
				
Disequilibrium (n = 19)	Falls	11%	32%	57%
	Gait examination	58% (Normal: 26%) (Abnormal:32%)	N/A	42%
	Neurological exam	68%	N/A	32%
	Romberg's sign	42% (Normal: 37%) (Abnormal: 5%)	N/A	58%
	Cerebellar signs	47% (Normal: 42%) (Abnormal: 5%)	N/A	53%
	Visual acuity exam	10% (Normal: 5%) (Abnormal: 5%)	N/A	90%
				
Others (n = 12)	Depression	0%	25%	75%
	Anxiety	25%	0%	75%

N/A: Not Applicable

As for the course of dizziness, only 2 patients have worsening symptoms (4%) and 60% of patients are referred to specialty services, the commonest ones of which are ENT (12%), neurology (8%) and cardiology (6%).

## Discussion

A striking finding from this study was that 46% of the patients did not have any diagnosis and 10% of them had more than one diagnosis. This finding is in accordance with the data from the review by Sloan et al [11] and illustrates the difficulty of diagnosing dizziness in a primary care setting. This is also reflected by a 40% referral rate to specialty services, which is higher compared to the 16% referral rate shown in a recent study [5]. On the other hand, the dizziness symptom worsened with time in only 4% of patients in this study, which is consistent with previous work [7] showing the generally "benign" course of this condition. The distribution of etiological causes of dizziness in this sample is also consistent with those of previous studies [7] with peripheral vestibular disorders (BPV and labyrinthitis) being the most common and accounting for 22% of diagnoses.

Effective history taking and communication between family physicians and patients is of crucial importance in the diagnosis of dizziness. The present chart audit study showed that family physicians were more likely to reach a diagnosis when patients' symptoms were documented in their own words, compared to those without such documentation.

Overall, the documentation rate of key quality indicators important to all dizziness subtypes were low, such as falls, syncope, symptoms of depression and anxiety, and orthostatic blood pressure changes. A history of falls is associated with increased morbidity but this was documented in only 36% of patients. This is especially worrying given that 28% of patients in our sample were known to be living alone. The finding that 60% of patients with dizziness are using 5 or more medications is consistent with Tinetti's population-based cross-sectional study [12].

Among vertiginous patients, the documentation rates for episode duration, relationship to head turning, hearing loss, and Hallpike maneuver were far from satisfactory. Among lightheaded patients, the documentation rates for symptom relationship to postural change, chest pain, palpitation, syncope, and orthostatic blood pressure changes are better but there is still room for improvement. Among patients with disequilibrium, the documentation rates for falls, gait examination, cerebellar signs, Romberg's sign and visual acuity examination were again sub-optimal. Among patients with non-specific dizziness, symptoms of depression and anxiety were also sub-optimally documented.

This study has several limitations. First, being a retrospective study, its strength is limited because there is no standardized strategy or protocol for data collection among different family physicians. Second, a chart audit is prone to documentation bias and incompleteness, depending on individual family physicians. In addition, the sample size of 50 is relatively small given the complexity of the clinical problem. Moreover, only one diagnostic billing code was used. Although this would catch the presenting symptom at its undifferentiated stage, the drawback is that we may underestimate the actual scope of the problem by ignoring patients who were coded more specifically by their dizziness subtypes. In addition, only one hospital site, namely Sunnybrook hospital, was selected for chart audit. Being an academic teaching center with predominantly older patients, the patient data from this hospital alone may not be generalizable to those of a community clinic setting.

Future directions for study would include the conduction of more prospective cohort studies on primary care patients using a standardized protocol for data collection This would assure uniform and consistent evaluation with the least amount of selection bias. Ideally, the use of inception cohorts would allow for better definition of the causes and natural history of dizziness in persons having their first episode. More prospective studies on dizziness outcomes with extended follow-up periods, as well as studies on different management strategies and specialty referral patterns for dizziness in the primary care setting would be helpful to family physicians. The ultimate goal is to identify the clinical and demographic or situational characteristics in a primary care setting that could help predict management decisions, such as diagnosing the most likely attributable cause of dizziness, delivering the most effective treatment for the symptom, and making the most timely and appropriate referral.

## Conclusions

Regardless of whether one regards dizziness as a "geriatric syndrome" or as a discrete "disease" for which a clear assessment algorithm could be established, there are certain quality indicators that are helpful for either approach but their documentation were found to be sub-optimal in this chart audit study. Allowing patients to describe their symptoms in their own words may help to improve diagnosis and management.

## Competing interests

The author(s) declare that they have no competing interests.

## Authors' contributions

EK contributed to the design and conception of the study, carried out the chart audit, analyzed the data and wrote the first draft of the manuscript. NP contributed to the design and conception of the study, supervised the conduct of the chart audit and data analysis and wrote and revised subsequent versions of the manuscript.

## Pre-publication history

The pre-publication history for this paper can be accessed here:



## Supplementary Material

Additional File 1 **1. Chart Audit Form **MS Word document with the chart audit form used in the study Click here for file
