# Supplementary material for: Assessment of dizziness among older patients at a family practice clinic: a chart audit study
Source: BMC Fam Pract. 2005 Jan 6;6:2. doi: 10.1186/1471-2296-6-2 (PMC546211; doi:10.1186/1471-2296-6-2)
Supplement: Additional File 1 — 1. Chart Audit Form MS Word document with the chart audit form used in the study [file 1471-2296-6-2-S1.doc]

**Additional File 1**

**Chart Audit Form**

Name: HFN: Family Dr: HSO/OHIP

Date of Birth:

Gender: Marital Status: Occupation: Ethnicity:

**Living situation:** alone/with spouse/with family/others

Date of First Encounter: Place of First Encounter:

Fam Dr Clinic/Walk-in Clinic/ER

Dates of Subsequent Followups:

Previous Episodes of Dizziness: Yes/No

If Yes, Date of First Episode/Presentation:

**Presentation:** Vertigo **Duration**:

Lightheadedness/Presyncope **Course**: Imbalance/unsteadiness/disequilibrium acute/episodic/chronic continuous

Others/Dizziness NYD If episodic:

More than 1 subtype: **Duration of each episode:** Frequency of episodes:

**Symptoms documented in Patient’s own words:** Yes:______________________________________

No

**Onset of symptom:** spontaneous precipitating factors: Postural change None Walking

Head turning Anxiety

Head or neck movement Micurition

Others

**Associated** Tinnitus Visual impairment

**Symptoms:** Hearing loss: Unilateral /Bilateral Hearing impairment

Ear fullness: unilateral/bilateral Recent febrile illness

Ear pain: unilateral/bilateral Recent head injury

Nausea/Vomitting Others:

Headache: migraine/ non-migraine None

Syncope/Blackout

Falls

CVS/Resp symptoms: Chest Pain/Palpitation/SOB

Focal Neurological symptoms: numbness/weakness/diplopia/dysarthria/others:

Symptoms of Anxiety/Panic attack

Symptoms of Depression

**Exacerbating/Relieving Factors:** Head movements

Standing up/Postural change

Urination

Exertion

Emotional stress

Others:

None

**Past medical History:**Previous episodes of vertigo/Lightheadedness/Disequilibrium

CAD/MI/AF/Arrythmia/Heart failure/Valvular heart disease

Stroke

HTN/ DM/ Hyperlipidaemia /Smoking

COPD/Asthma

Vestibular disorders/Meniere’s disease/Chronic OM/cholesteatoma/others

Panic attack/Anxiety disorder /Depression /Other psychiatric disease

Dementia

Hx of Falls

Hx of Head Trauma

Migraine Headache

Herpes zoster

Neurological:Multiple sclerosis/CNS tumour/Parkinsons/Seizures/others

Vitamin B12 deficiency/ Thyroid disease

Osteoarthritis/Other arthritis

Osteopenia/Osteoporosis/Fractures

GERD/Heartburn/Dyspepsia/PUD

Visual impairment: Cataract/Glaucoma/others

Hearing impairment

Alcohol use Others: None

Past Surgery

**Family History:** Porphyria/Amyloidosis/CVS diseases

Others None

**Medications:** Sedatives/ Antidepressants

Antihypertensives

DM medications: oral hypoglycemics/insulin

Anticholinergics

Ototoxic Medications: aminoglycosides/others

Antiplatelet agents/anticoagulants

Lipid lowering agents

Thyroxine

Hormones replacement/Bisphosphonates

Asthma Puffers

Steroids

NSAIDS Others:

None

**Physical signs:**

Vitals: BP Pulse Temp RR O2sat

Alert Orientated

Orthostatic vital signs: BP drop: Pulse increase:

Postural dizziness

Otoscopic exam: Tympanic membrane: normal/abnormal

External ear canal: vesicles

Weber/Rhinne test

Fundoscopic exam: cataract/macular degeneration

Head and Neck: Cervical spine

CVS: carotid bruit/ HS: (Normal/Abnormal) /heart murmurs

Resp:

Rectal exam for OB

Neuro: Nystagmus: Spontaneous/Gaze evoked

Unidirectional/multidirectional

Direction: Torsional/Vertical/Horizontal

Cranial Nerves exam: Pupils/EOM/Gag/Facial symmetry/Others

Gait

Sensory exam

Motor exam

Reflexes: Deep tendon/Babinski

Cerebellar exam

Romberg testing

Hearing

Visual acuity

Hallpike maneuver: Positive: Peripheral Negative

Central

Hyperventilation (3 min)

Minimental Status exam: score- Psychiatric: Mental Status exam

Others:

**Investigations:** Routine Labs: CBC, ESR,BUN,Cr,lytes,random glucose

Cardiac enzymes

Fasting Cholesterol profile

LFT TFT

12 lead ECG

Holter monitoring

Carotid doppler

Echocardiogram/Exercise Stress test/Sestamibi scan

Audiometry

Vestibular testing/ENG/tilt testing

CT /MRI

Others:

None

**Treatment given:**

Medications: Vestibular sedatives

Diuretics

Antidepressants/antianxiety drugs

Others:

Reduction of polypharmacy/Discontinuing medication

Counseling on Safety issues: Falls /Driving

Canalith repositioning procedure/Epley Manuovre

Low salt diet

Referral:Geriatrician

Otolaryngologist/Dizziness Clinic at Sunnybrook

General Internist

Cardiologist

Ophthalmologist

Others:

Physiotherapy/Occupational therapy/Home care

Follow up: Family Dr/Specialist

Admission

Others:

None

**Diagnosis:** Yes: More than 1:

No

**Outcome:** Resolved/Improved/Unchanged/worsened/unknown(not documented)/others:

**Remarks/Comments:**
